# MicroRNA-497 induced by *Clonorchis sinensis* enhances the TGF-β/Smad signaling pathway to promote hepatic fibrosis by targeting *Smad7*

**DOI:** 10.1186/s13071-021-04972-3

**Published:** 2021-09-14

**Authors:** Qian-Yang Zhou, Hui-Min Yang, Ji-Xin Liu, Na Xu, Jing Li, Li-Ping Shen, Yu-Zhao Zhang, Stephane Koda, Bei-Bei Zhang, Qian Yu, Jia-Xu Chen, Kui-Yang Zheng, Chao Yan

**Affiliations:** 1 Jiangsu Key Laboratory of Immunity and Metabolism, Department of Pathogenic Biology and Immunology, Xuzhou Key Laboratory of Infection and Immunity, Xuzhou Medical University, Jiangsu 221004 Xuzhou, People’s Republic of China; 2 National Experimental Demonstration Center for Basic Medicine Education, Department of Clinical Medicine, Xuzhou Medical University, Xuzhou, 221004 Jiangsu People’s Republic of China; 3 Department of Dermatology, The Second Affiliated Hospital of Nanjing Medical University, Nanjing, 210011 Jiangsu China; 4 National Institute of Parasitic Diseases, Chinese Center for Disease Control and Prevention, Key Laboratory of Parasite and Vector Biology, Ministry of Health, WHO Collaborating Center of Malaria, Schistosomiasis and Filariasis, Shanghai, 200025 People’s Republic of China

**Keywords:** miR-497, Smad7, TGF-β/Smad signaling pathway, Hepatic fibrosis, ESPs of *C. sinensis*

## Abstract

**Background:**

Various stimuli, including *Clonorchis sinensis* infection, can cause liver fibrosis. Liver fibrosis is characterized by the activation of hepatic stellate cells (HSCs) with massive production of extracellular matrix (ECM). Our previous study showed that the TGF-β_1_-induced Smad signaling pathway played a critical role in the activation of HSCs during liver fibrosis induced by worm infection; however, the mechanisms that modulate the TGF-β/Smad signaling pathway are still poorly understood. Accumulating evidence demonstrates that miRNAs act as an important regulator of activation of HSCs during liver fibrosis.

**Methods:**

The target of miR-497 was determined by bioinformatics analysis combined with a dual-luciferase activity assay. LX-2 cells were transfected with miR-497 inhibitor and then stimulated with TGF-β_1_ or excretory/secretory products of *C. sinensis* (CsESPs), and activation of LX-2 was assessed using qPCR or western blot. In vivo, the mice treated with CCl_4_ were intravenously injected with a single dose of adeno-associated virus serotype 8 (AAV8) that overexpressed anti-miR-497 sequences or their scramble control for 6 weeks. Liver fibrosis and damage were assessed by hematoxylin and eosin (H&E) staining, Masson staining, and qPCR; the activation of the TGF-β/Smad signaling pathway was detected by qPCR or western blot.

**Results:**

In the present study, the expression of miR-497 was increased in HSCs activated by TGF-β_1_ or ESPs of *C. sinensis*. We identified that *Smad7* was the target of miR-497 using combined bioinformatics analysis with luciferase activity assays. Transfection of anti-miR-497 into HSCs upregulated the expression of Smad7, leading to a decrease in the level of p-Smad2/3 and subsequent suppression of the activation of HSCs induced by TGF-β_1_ or CsESPs. Furthermore, miR-497 inhibitor delivered by highly-hepatotropic (rAAV8) inhibited TGF-β/smads signaling pathway by targeting at Smad7 to ameliorate CCL4-induced liver fibrosis.

**Conclusions:**

The present study demonstrates that miR-497 promotes liver fibrogenesis by targeting *Smad7* to promote TGF-β/Smad signaling pathway transduction both in vivo and in vitro, which provides a promising therapeutic strategy using anti-miR-497 against liver fibrosis.

**Graphical Abstract:**

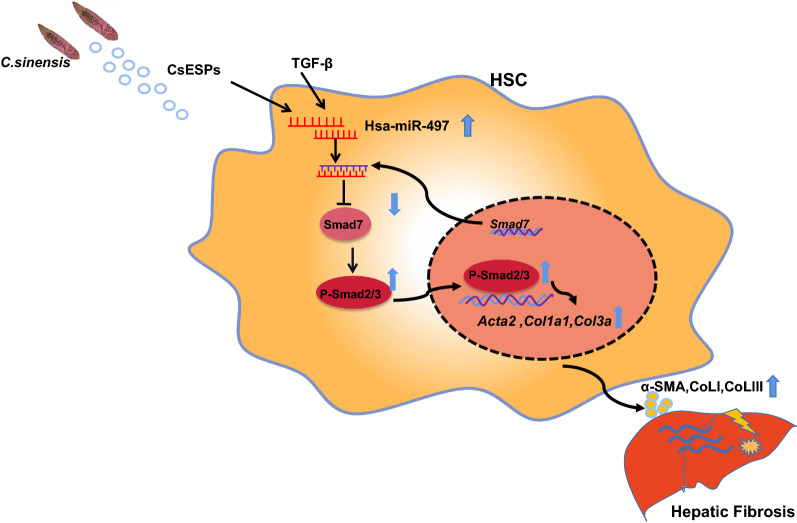

## Background

*Clonorchis sinensis* is a key food-borne parasite that can be ingested by the consumption of undercooked or raw fresh fish containing metacercaria. The worm is widely distributed in eastern Asia, including China, Korea, and Vietnam, and more than 15 million people are thought to be infected [1]. *C. sinensis* was classified as a group 1 carcinogen by the International Agency for Research on Cancer [2]. The worms dwell in the bile duct and cause cholangitis, cholestasis, and progressively hepatic fibrosis (cirrhosis) [3]. Hepatic fibrosis caused by infection with *C. sinensis* is characterized by excess deposition of extracellular matrix (ECM), which can ultimately lead to biliary dysfunction, cirrhosis, hepatocellular/biliary carcinoma, and liver failure if there is no proper intervention [4]. However, the understanding of the hepatic fibrogenesis caused by *C. sinensis* is still incomplete.

Activated hepatic stellate cells (HSCs) are the main contributor to the production of ECM [5]. HSCs are quiescent in normal conditions; however, if there is an insult, the HSCs can differentiate into a myofibroblast-like phenotype that is contractile, proliferative, and fibrogenic [6]. The activation of HSCs is integrated through complex signaling networks that regulate the deposition of the extracellular matrix, of which the TGF-β/Smad signaling pathway orchestrates the activation of HSCs and plays a critical role in the development of hepatic fibrosis [7–9]. The TGF-β signaling pathway plays a critical role in the progression of liver fibrosis by regulating activation of HSCs and the massive production of ECM [10]. Mechanistically, TGF-β_1_ binds to TGF-β type I (TGF-βRI) and type II (TGF-βRII) receptors which activate and phosphorylate Smad2/3(P-Smad2/3), leading to a subsequent interaction with Smad4 [11]. The Smad2/3/4 complex can then translocate to the nucleus and induce the expression of profibrotic genes, namely collagen type I [12]. During the process, Smad7 as a potent negative regulator can compete with Smad2 to bind the MH2 TGF-βRI receptor (T beta R-I), thus inhibiting Smad2 phosphorylation and subsequent signaling transduction [13].

Previous studies have shown that microRNAs are involved in cell proliferation, differentiation, programming, apoptosis, and cell death in tissues and organs, and are closely related to the occurrence of a variety of human diseases [14, 15]. It has also been reported that miRNAs are involved in helminth infection as well as the fibrosis caused by the infection [16]. For example, decreased miR-27b can ameliorate *Schistosoma japonicum*-caused liver fibrosis by the upregulation of KH-type splicing regulatory protein (KSRP), and KSRP can promote stabilization of TGF-β_1_ [17]. Mmu-miR-92a-2-5p can decrease liver fibrosis caused by *S. japonicum* infection [18]. However, little is known about the relational roles of miRNAs in the biliary fibrosis caused by *C. sinensis*.

A growing body of evidence indicates that miRNAs are involved in the regulation of liver fibrosis by targeting the TGF-β signaling pathway during the activation of HSCs [19–21]. Our previous studies showed that Smad7 was abnormally expressed in the liver of mice infected with *C. sinensis*, and we found that many miRNAs were involved in regulating the progression of liver fibrosis caused by *C. sinensis* at the post-transcriptional level [22, 23]. However, the mechanisms by which miRNAs regulate TGF-β/Smad to promote the activation of HSCs remain obscure. Our previous study also found that miR-497 was significantly increased in liver fibrosis caused by *C. sinensis* and correlated negatively with Smad7, which suggested that miR-497 has a potential regulatory role in biliary fibrosis caused by *C. sinensis*, but the underlying mechanisms have not been addressed [22]. Given this background, we used human hepatic stellate LX-2 cells to investigate the roles and mechanisms of miR-497 in the activation of LX-2 cells to promote liver fibrosis. In our present study, we found that miR-497 facilitated the TGF-β/Smad signaling pathway by targeting *Smad7*, which promoted the activation of HSCs and exacerbated liver fibrosis in vivo and in vitro, respectively. Our data provide a potential therapeutic implication for intervention in liver fibrosis.

## Methods

### Ethics

Animal care and all experiments in this study were carried out under the guidelines of the National Laboratory Animal Center. The main procedures and protocol were approved by the Animal Care and Use Committee of Xuzhou Medical University (license 201501w002).

### Preparation of *C. sinensis* excretory/secretory products

Excretory/secretory products from adult *C. sinensis* (CsESPs) were prepared as described elsewhere [24]. In brief, 8-week-old white guinea pigs were individually infected with 200 metacercariae of *C. sinensis*. The animals were euthanized at 8 weeks post-infection under deep anesthesia with ethyl ether, and the livers were extracted. Adult worms were then collected from the bile ducts and washed five times with sterile phosphate-buffered saline (PBS) containing 1% penicillin/streptomycin (Beyotime, Shanghai, China), followed by incubation for 24 h at 37 °C with 5% CO_2_. After incubation, the medium was collected and centrifuged for 10 min at 1000×*g* to remove any cellular debris. The supernatant was then centrifuged for a further 10 min at 18,000×*g* before filtering with a syringe-driven 0.45 μm filter. The concentration of protein was measured using a BCA protein concentration determination kit (Beyotime, Shanghai, China) and stored at −80 °C for further use.

### miR-497 target prediction

TargetScan (http://www.targetscan.org) [25], MiRanda (http://www.microrna.org/microrna/home.do) [26], and PicTar (http://pictar.mdc-berlin.de/) [27] were used to predict possible target genes of miR-497 and conserved sites across different species bound by the seed region of miR-497 in silico.

### Cell culture

A well-characterized human HSC cell line, LX-2, was commercially available from Xiangya Medical College Biomedical Center. The cells were cultured and maintained as described elsewhere [8]. For TGF-β_1_ and CsESPs stimulation experiment, the cells were firstly starved for 12 h in Dulbecco’s modified Eagle medium (DMEM) without 10% fetal bovine serum (FBS), and then the cells maintained in DMEM with 10% FBS were stimulated using TGF-β_1_ (12 ng/ml) or ESPs (60 μg/ml) for 48 h. The cells were harvested and stored in TRIzol for quantitative real-time polymerase chain reaction (qRT-PCR) assay.

### Transfection of miR-497 inhibitor and mimics

The miR-497 inhibitor, miR-497 mimics, and negative (scramble) control (120 pmol/50 µl) were purchased from Guangzhou RiboBio Co., LTD (Guangzhou, China), and sequences are shown in Table 1.

**Table 1:** The primers used in the present study.

Target	Oligonucleotide sequence (5'–3′)	
	Forward primer	Reverse primer
Has-*β-actin*	GCCCTGAGGCACTCTTCCA	TTGCGGATGTCCACGTCA
Has-*Col1a1*	ACTGGTGAGACCTGCGTGTA	AATCCATCGGTCATGCTCTC
Has-*Smad7*	CCCCATCACCTTAGCCGACTCTGC	CCCAGGGGCCAGATAATT
Has-*miR-497*	CAGCAGCACACTGTGGTTTG	
Has-*Acta2*	TTCATCGGGATGGAGTCTGCTGG	TCGGTCGGCAATGCCAGGGT
*U6*	ATGGGTCGAAGTCGTAGCC	TTCTCGGCGTCTTCTTTCTCG
mmu-*miR-497*	CAGCAGCACACTGTGGTTTGTA	
mmu-*Smad7*	AGAGGCTGTGTTGCTGTGAATC	CCATTGGGTATCTGGAGTAAGGA
mmu-*β-actin*	AACTCCATCATGAAGTGTGA	ACTCCTGCTTGCTGATCCAC
mmu-*Col1a1*	TAGGCCATTGTGTATGCAGC	ACATGTTCAGCTTTGTGGACC

For the miR-497 inhibition experiment, LX-2 cells were first starved for 12 h in DMEM without 10% FBS at 37 °C in a humidified chamber supplemented with 5% CO_2_ and then were transfected with miR-497 inhibitor (2.4 μmol/ml) or negative control (2.4 μmol/ml) using Lipofectamine 2000 (Thermo Fisher Scientific, Waltham, MA, USA) for 6 h before TGF-β_1_ (15 ng/ml) or CsESPs stimulation according to the manufacturer’s instructions. LX-2 cells were further maintained in the stimulation with TGF-β_1_ (15 ng/ml) in DMEM containing 10% FBS for 48 h. For the overexpression of miR-497, LX-2 cells that were starved for 12 h were transfected with miR-497 mimics (2.4 μmol/ml) or control (2.4 μmol/ml) by Lipofectamine 2000 ( Thermo Fisher Scientific, Waltham, MA, USA) for 48 h in DMEM. The cells were then collected and stored in TRIzol at −80 °C for qRT-PCR.

### Dual-luciferase activity assay

A total of 293T cells were kindly gifted by Prof. Feng Zhou (Xuzhou Medical University). The cells were cultured in DMEM supplemented with 10% FBS (Gibco, NY, USA). Cells were cultivated at 37 °C in a humidified chamber supplemented with 5% CO_2_. The *Smad7* 3′-UTR reporter plasmid (GENERAY, Shanghai, China) (*Smad7* 3′-UTR Wt) and *Smad7* 3′-UTR reporter plasmid with a mutant at the miR-497 binding site (*Smad7* 3′-UTR Mut) were purchased from Creative Biogene (Shirley, NY, USA). HSCs (5 × 10^4^ cells per well) were co-transfected with pmiRGLO-empty vector (16 μg/ml), *Smad7* 3′-UTR Wt (16 μg/ml), or *Smad7* 3′-UTR Mut (16 μg/ml) with miR-497 mimics (800 pmol/ml) by lipofectamine 2000 (Invitrogen, MA, USA) for 48 h according to the manufacturer’s recommendation. Cells were collected and assayed using the dual-luciferase reporter assay system gene assay kit (Promega, WI, USA) according to the manufacturer’s instructions.

### Animal model of CCl_4_-induced liver fibrosis

Male BALB/c mice (*n* = 18; 6 weeks old; weight, 22–25 g) were obtained from the Experimental Animal Center of Xuzhou Medical University (Xuzhou, China) and were kept in a standard laboratory in an air-conditioned room with free access to food and water. All experimental protocols were conducted according to the Guide for the Care and Use of Laboratory Animals and were approved by the Ethics Review Board for Animal Studies of Xuzhou Medical University.

Liver fibrosis was induced by injection of CCl_4_ as described previously, with slight modifications. Specifically, 12 mice were treated with CCl_4_ (diluted 1:5 in olive oil, 5 µl/g) injected intraperitoneally twice weekly for 6 weeks. For the normal control group, six mice were injected intraperitoneally with the same volume of olive oil. At the same time, the mice in the two CCl_4_-treated groups were intravenously injected with 1 × 10^12^ adeno-related viruses that overexpressed anti-miR-497 sequences or their scramble control (manufactured by GENECHEM, Shanghai, China). Following 6 weeks of the treatment with CCl_4_ or olive oil, all the mice from each group were sacrificed by euthanasia. The livers and sera from each mouse were harvested for further experiments.

### Detection of alanine aminotransferase (ALT) activity

The activity of ALT in sera from mice was detected using an ALT/GPT test kit (Jiancheng Institute of Biotechnology, Nanjing, China). The levels were analyzed spectrophotometrically according to the manufacturer's instructions.

### Hematoxylin and eosin (H&E) staining, Masson staining, and Sirius red staining

For histological analysis, liver tissues were excised and fixed with 4% paraformaldehyde for 24 h. Thereafter, the fixed tissues were embedded in paraffin, sliced to a thickness of 4 µm, and routinely stained with H&E, Masson staining, or Sirius red staining according to the manufacturer’s recommendations, and the pathology score of H&E staining was evaluated [28].

### Detection of hepatic hydroxyproline (HYP) content

HYP content was determined using a commercially available kit (Jiancheng Institute of Biotechnology, Nanjing, China) according to the manufacturer's recommendations.

### RNA extraction and qRT-PCR analysis

Total RNA from cells or partial liver of mice was extracted using TRIzol reagent (Thermo Fisher Scientific, MA, USA), following the manufacturer’s instructions, and then reverse-transcribed into cDNA using M-MLV reverse transcriptase (Thermo Fisher Scientific, MA, USA) or the Hairpin-it miRNAs qRT-PCR Quantitation Kit (GenePharma, Shanghai, China). Then, qRT-PCR was performed using the SYBR Green Master Mix and run on a real-time PCR system (Roche, Basel, Switzerland). The relative expression levels of miRNAs or mRNAs were normalized to U6 small nuclear RNA (snRNA) or β-actin following the 2^−△△^ Ct comparative method, respectively. The primers are listed in Table 1.

### Western blot analysis

Cells or liver homogenates were harvested and washed twice in cold PBS and then were treated with RIPA lysis buffer (Beyotime, Shanghai, China) on ice for 30 min. The lysate was collected into micro-tubes and centrifuged for 15 min at 12,000 rpm at 4 °C. Protein samples (20 mg) were denatured with 5× SDS loading buffer at 100 °C for 5 min, and then were segregated on 10% SDS polyacrylamide gel electrophoresis and transferred onto 0.45-mm nitrocellulose membranes. The membranes were cut into bands of appropriate width according to the protein marker and after 60 min of blocking with 5% fat-free milk, membranes were incubated with Smad 7 antibody (1:2000; Sigma, USA), COLI antibody (1:2000; Sigma, USA), P-Smad2/3 antibody (1:1000; Abclonal, Wuhan, China), COLIII antibody (1:1000, Abclonal, Wuhan, China), α-SMA (1:1000, Abclonal, Wuhan, China), and β-actin antibody (1:2000; Abmart, Shanghai, China) overnight at 4℃, correspondingly. Blots were washed for 1 h with the anti-rabbit secondary antibody (1:2000; Cell Signaling Technology, USA). After washing three times with TBST, immunoreactive protein bands were detected using enhanced chemiluminescence reagents (Bio-Rad, CA, USA). Band intensities were normalized to β-actin and analyzed using ImageJ software.

### Statistical analysis

All data obtained from at least three independent experiments are presented as means ± standard error (SE). The statistical analysis was performed using the SPSS version 19.0 software package. Differences among more than two groups were assessed by one-way analysis of variance (ANOVA) followed by the least significant difference (LSD) test unless otherwise stated. If appropriate, a two-tailed Student *t*-test was used to assess differences for comparison of two groups. A *P*-value less than 0.05 was considered to be statistically significant.

## Results

### Upregulated expression of miR-497 in HSCs is activated by *C. sinensis* ESPs and TGF-β_1_

**Fig. 1:**
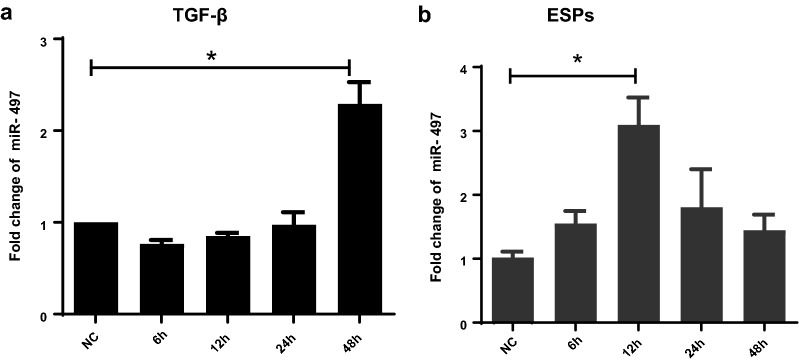
The expression patterns of miR-497 in TGF-β_1_- or CsESPs-activated HSCs.** a** Relative expression of miR-497 in LX-2 cells stimulated by TGF-β_1_ (12 ng/ml) for 6, 12, 24, and 48 h was determined using qRT-PCR. **b** Relative expression of miR-497 in LX-2 cells stimulated by CsESPs of *C. sinensis* (60 μg/ml) for 6, 12, 24, and 48 h was determined using qRT-PCR. *P < 0.05, compared with the indicated group. All the data represent at least three independent experiments

To investigate the expression of miR-497 in the activated HSCs, the LX-2 cells were stimulated by TGF-β_1_ or ESPs of *C. sinensis* (CsESPs) for 6, 12, 24, and 48 h. The expression dynamics of miR-497 in LX-2 cells activated by TGF-β_1_ or CsESPs were detected by qRT-PCR (Fig. 1). The results showed that the expression of miR-497 in LX-2 cells stimulated by TGF-β_1_ was higher at 48 h than that in normal control cells (Fig. 1a, 48 h: *t*_(5)_ = −5.42, *P* = 0.026). However, in CsESPs-stimulated HSCs, qRT-PCR data showed that miR-497 was significantly upregulated at 12 h compared with the normal control group (Fig. 1b, 12 h: *t*_(5)_ = −4.706, *P* = 0.007). These data suggest that miR-497 was significantly upregulated in activated HSCs.

### *Smad7* is the target of miR-497

**Fig. 2:**
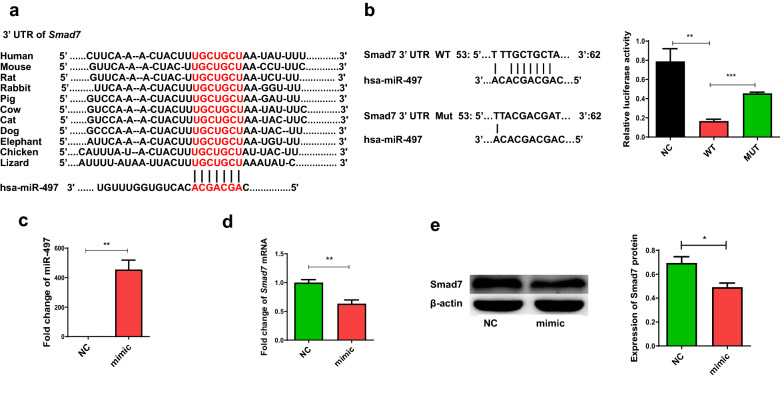
*Smad7* is the direct target of miR-497. **a** Bioinformatics analysis as predicted by TargetScan, miRanda, miRDB, and miRWalk showed that miR-497 has binding sites in the *Smad7* 3′ UTR of different species. **b** Relative firefly luciferase activity in 293 T cells that were co-transfected with pmiRGLO-empty vector (16 μg/ml), *Smad7* 3′-UTR WT (16 μg/ml), or *Smad7* 3′-UTR MUT (16 μg/ml) with miR-497 mimics (800 pmol/ml) using Lipofectamine 2000. Overexpression of miR-497 in LX-2 cells suppressed the expression of *Smad7*. LX-2 cells were transfected by miR-497 mimics for 48 h, and the cells were harvested. The relative expression of miR-497 (**c**), *Smad7* mRNA transcripts (**d**), and Smad7 protein (**e**) were determined. *P < 0.05, **P < 0.01, *** *P* < 0.001, compared with the indicated group. All the data represent at least three independent experiments

Bioinformatics analysis indicated that the 3′-UTR of *Smad7* contained the binding site of miR-497, and the binding site is highly conserved across many different species (Fig. 2a). To further clarify whether miR-497 can inhibit the expression of Smad7 by binding to the 3′ UTR of *Smad7*, we constructed the luciferase reporter plasmid pmiRGLO with the 3′ UTR of *Smad7* containing the miR-497 binding site on the 3′ UTR of *Smad7* (WT plasmid), as well as luciferase reporter with *Smad7* 3′ UTR containing the miR-497 binding site mutation (MUT plasmid). The data showed that luciferase activity in the miR-497 mimic group was decreased compared with that in the no-load plasmid control group, with a statistically significant difference (Fig. 2b, NC vs. WT: *F*_(3, 12)_ = 16.085, *P* = 0.0021). When MUT plasmid was co-transfected into 293T cells with miR-497 mimics, the activity of the reporter gene showed no difference from the non-load plasmid control group, but increased compared with the wild-type plasmid group, with a statistically significant difference (Fig. 2b, WT vs. Mut: *F*_(3, 12)_ = 16.085, *P* < 0.001). To determine whether miR-497 has a regulatory role in the expression of Smad7, we transfected miR-497 mimic into LX-2 cells for 48 h, and a statistically significant increase in the relative expression level of miR-497 compared with the control group was observed (Fig. 2c, *t*_(3)_ = 7.082, *P* = 0.0021), suggesting that miR-497 was successfully transfected into LX-2 cells. Furthermore, the levels of *Smad7* mRNA transcript were decreased in LX-2 after treatment with the miR-497 mimic (Fig. 2d, *t*_(3)_ = 4.437, *P* = 0.0091). Similarly, the level of Smad7 in the miR-497 mimic treated with LX-2 was lower than that in the scramble control-treated cells (Fig. 2e, *t*_(3)_ = 3.192, *P* = 0.0332). Taken together, these data indicated that miR-497 directly inhibits the expression of Smad7 in HSCs.

### Inhibition of miR-497 suppresses the TGF-β signaling pathway by targeting *Smad7*

**Fig. 3:**
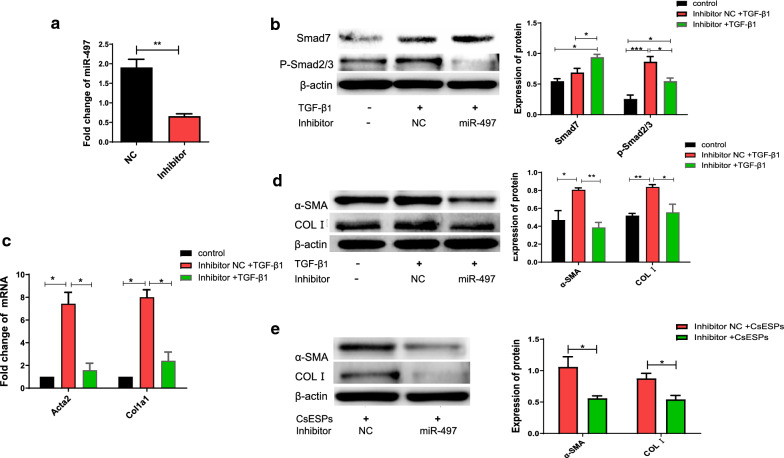
The TGF-β/Smad signaling pathway is regulated by miR-497 via targeting of *Smad7.* LX-2 cells were transfected with miR-497 inhibitor as well as its scramble and then stimulated by TGF-β_1_ for 48 h, and the cells were harvested for qRT-PCR and western blot. **a** The relative levels of mature miR-497 were determined by qRT-PCR. **b** The expression of Smad7 and the down-stream p-Smad2/3 were detected by western blot. **c**, **d** The activation of HSCs was evaluated by the levels of *Acta2* (encoding α-SMA) and *Col1a1* (encoding COLI) using both qRT-PCR and western blot, respectively. **e** The levels of α-SMA and collagen I (COLI) production using western blot in LX-2 cells transfected with miR-497 inhibitor as well as its scramble and then stimulated by CsESPs for 48 h. *P < 0.05, **P < 0.01, ***P < 0.001, compared with the indicated group. All the data represent at least three independent experiments

To further verify whether miR-497 may be involved in TGF-β_1_-induced LX-2 cell activation by regulating the expression of Smad7, LX-2 cells were transfected with miR-497 inhibitor as well as its scramble and then stimulated by TGF-β_1_ for 48 h. The results showed that, compared with the scramble group, the relative expression of miR-497 was markedly reduced after transfection with miR-497 inhibitor, and the difference was statistically significant (Fig. 3a, *t*_(3)_ = 5.766, *P* = 0.0045). Furthermore, the transfection of miR-497 inhibitor significantly increased the expression of Smad7, compared with the scrambled control (Fig. 3b, inhibitor + TGF-β_1_ vs. inhibitor NC + TGF-β_1_: *F*_(3, 12)_ = 13.321, *P* = 0.002; inhibitor + TGF-β_1_ vs. control: *F*_(3, 12)_ = 13.321, *P* = 0.017); subsequently, p-Smad2/3 protein in transfection of miR-497 inhibitor-HSCs was considerably decreased (Fig. 3b, control vs. inhibitor NC + TGF-β_1_: *F*_(3, 12)_ = 13.321, *P* = 0.002; control vs. inhibitor + TGF-β_1_: *F*_(3, 12)_ = 13.321, *P* = 0.017). Furthermore, the expression of collagen I (encoded by *Col1a1*) and α-SMA (encoded by *Acta2*) was also decreased in TGF-β_1_-stimulating LX-2 cells which were pretreated with miR-497 inhibitor, compared with that in non- or scramble-transfected LX-2 cells at both the mRNA and protein levels (Fig. 3c and Fig. 3d, *Col1α1*: *F*_(3, 12)_ = 39.932 27.621, *P* = 0.001; *Acta*: *F*_(3, 12)_ = 32 27.621, *P* = 0.001; α-SMA: *F*_(3, 12)_ = 9.962, *P* = 0.006; C: *F*_(3, 12)_ = 9.343, *P* = 0.013).

As our previous study showed that the expression of miR-497 was significantly increased in the liver of *C. sinensis*-infected mice and that CsESPs activated the TGF-β/Smad signaling pathway to promote activation of HSCs [7, 22], we further investigated whether miR-497 was involved in the CsESP activation of HSCs or not. The data showed that the specific miR-497 inhibitor significantly decreased the expression of α-SMA and COLI in the activation of HSCs induced by CsESPs compared with the scramble control-treated groups, indicating that miR-497 promoted the activation of HSCs induced by CsESPs (Fig. 3e, α-SMA: *t*_(3)_ = 2.996, *P* = 0.041; COLI: *t*_(3)_ = 3.227, *P* = 0.032). Collectively, these data demonstrate that the downregulation of miR-497 expression reduced the activation of HSCs via repressing the TGF-β/Smad signaling pathway.

### Inhibition of miR-497 in mice reduced CCl_4_-induced liver damage

**Fig. 4:**
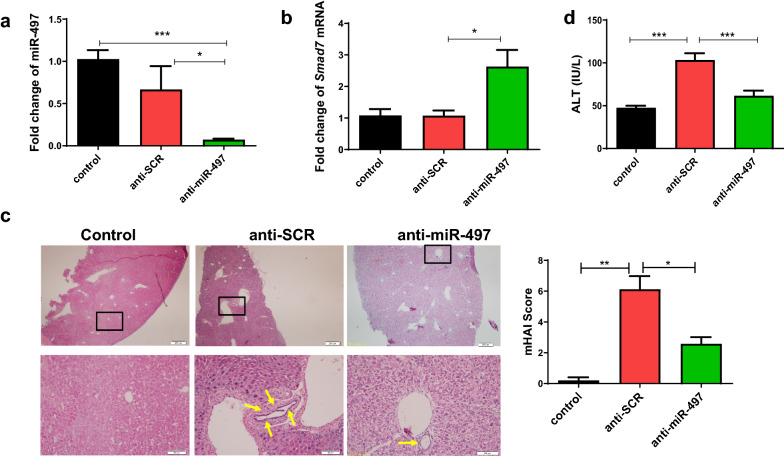
Inhibition of miR-497 in mice reduced carbon tetrachloride (CCl_4_)-induced liver damage. A mouse model of liver fibrosis was established by injection of CCl_4_ for 6 weeks, and the mice were treated with highly hepatotropic rAAV8-anti-miR-497 (anti-miR-497) or control rAAV8-scramble vectors (anti-SCR) with a single dose of 1 × 10^12^ virus genomes or PBS by tail vein injection onset of CCl_4_ injection. **a** The relative expression of miR-497 in the livers of mice from different groups. **b** The relative expression of *Smad7* in the livers of mice from different groups. **c** Histological analysis of liver injuries by H&E staining in indicated groups. **d** The activities of ALT in the sera from each mouse were determined using commercial kits. *P < 0.05, **P < 0.01, *** *P* < 0.001, compared with the indicated group. All data represent at least three independent experiments

To investigate whether inhibition of miR-497 has a therapeutic effect on CCl_4_-induced liver damage in vivo, we injected carbon tetrachloride (CCl_4_) for 6 weeks to establish a mouse model of liver fibrosis, and the mice were treated with highly hepatotropic rAAV8 anti-miR-497 (anti-miR-497) or control rAAV8-scramble vectors (anti-SCR) with a single dose of 1 × 10^12^ virus or PBS by tail vein injection onset of CCl_4_ injection. Firstly, we found that the level of mature miR-497 in the liver of anti-miR-497 mice was remarkably decreased compared with that of the anti-SCR or PBS group (Fig. 4a, anti-miR-497 vs. anti-SCR: *F*_(3, 18)_ = 7.959, *P* < 0.001; anti-miR-497 vs. PBS: *P* = 0.016). As demonstrated above, miR-497 can target *Smad7* to regulate the expression of Smad7. Therefore, we further determined the level of *Smad7* using qRT-PCR, which revealed a more than twofold increase in *Smad7* expression in the liver of anti-miR-497-transfected mice compared with the anti-SCR mice (Fig. 4b, F_(3, 18)_ = 7.008, *P* = 0.017).

H&E staining revealed disordered arrangement of the hepatic sinusoid, extensive hepatocellular degeneration and necrosis, and inflammatory cell infiltration in anti-SCR CCl_4_ mice; however, these histological changes were ameliorated after treatment with rAAV8-anti-miR-49 lentivirus (Fig. 4c), and pathology scores are statistically significant (Fig. 4c, control vs. anti-SCR: *F*_(3, 18)_ = 30.892, *P* = 0.004; anti-SCR vs. anti-miR-497, *F*_(3, 18)_ = 30.892, *P* = 0.013). The levels of the serum activities of ALT in the mice of the anti-miR-497 group were significantly reduced compared with those in the anti-SCR group, suggesting that hepatic damages caused by CCl_4_ were alleviated by inhibiting miR-497 (Fig. 4d, control vs. anti-SCR: *F*_(3, 18)_ = 23.591; anti-SCR vs. anti-miR-497, *F*_(3, 18)_ = 23.591, *P* < 0.001, *P* < 0.001).

### Inhibition of miR-497 in mice reduced CCl_4_-induced liver fibrosis by targeting *Smad7*

**Fig. 5:**
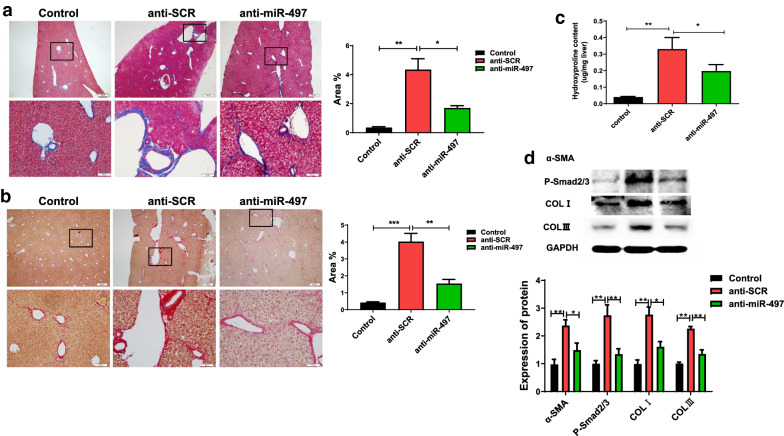
Inhibition of miR-497 in mice reduced CCl_4_-induced liver fibrosis. **a** Collagen deposition shown by Masson staining in the livers from different groups as indicated. **b** Sirius red staining showed collagen deposition in the livers from different groups as indicated. **c** Hydroxyproline (HYP) content in the liver of mice in each group as indicated. **d** Levels of α-SMA, P-Smad2/3, COLI, and COLIII in in the livers from different groups determined by western blot. *P < 0.05, **P < 0.01, compared with the indicated group. All data represent at least three independent experiments

Next, we investigated the protective roles of anti-miR-497 in liver fibrosis caused by CCl_4_. Masson staining showed obvious collagen deposition in the portal and sinusoidal areas in the CCl_4_ mouse model group; however, rAAV8 anti-miR-49 lentivirus resulted in a decrease in collagen deposition (Fig. 5a, control vs. anti-SCR: *F*_(3, 12)_ = 21.489, *P* = 0.002; anti-SCR vs. anti-miR-497: *F*_(3, 12)_ = 21.489, *P* = 0.048). We further detected collagen deposition in the liver using Sirius red staining. Similar to Masson staining, it showed that collagen deposition in the liver of rAAV8 anti-miR-49 lentivirus-treated mice was significantly decreased compared with that in scrambled lentivirus-treated (anti-SCR) mice (Fig. 5b, control vs. anti-SCR: *F*_(3, 12)_ = 33.112, *P* < 0.001; anti-SCR vs. anti-miR-497: *F*_(3, 12)_ = 33.112, *P* = 0.004). As hydroxyproline (HYP) content is a characteristic of liver fibrosis, we examined HYP levels in each group. The data showed a significant decrease in HYP content in the rAAV8-anti-miR-497-transfected mice that were subjected to CCl_4_ injection compared with the rAAV8-anti-SCR group (Fig. 5c, control vs. anti-SCR: *F*_(3, 15)_ = 7.941, *P* = 0.002; anti-SCR vs. anti-miR-497: *F*_(3, 15)_ = 7.941, *P* = 0.044). Furthermore, we examined the expression of an α-SMA-a marker of hepatic fibrosis by western blot; the data showed that the level of α-SMA was both significantly ameliorated in CCl_4_ mice treated with rAAV8-anti-miR-497, and Smad7 downstream p-Smad2/3 protein was also considerably decreased compared with rAAV8 anti-SCR (Fig. 5d, α-SMA: control vs. anti-SCR: *F*_(3, 12)_ = 9.910, *P* = 0.002; anti-SCR vs. anti-miR-497: *F*_(3, 12)_ = 9.910, *P* = 0.027; p-Smad2/3: control vs. anti-SCR: *F*_(3, 12)_ = 9.910, *P* = 0.002; anti-SCR vs. anti-miR-497: *F*_(3, 12)_ = 9.910, *P* = 0.004). We also examined other fibrosis markers including COLI and COLIII using western blot. The results showed that both were significantly decreased in CCl_4_ mice treated with rAAV8-anti-miR-497, compared with the mice that were treated with rAAV8 anti-SCR (Fig. 5d, COLI: control vs. anti-SCR: *F*_(3, 12)_ = 11.922, *P* = 0.001; anti-SCR vs. anti-miR-497: *F*_(3, 12)_ = 11.922, *P* = 0.018. COLIII: control vs. anti-SCR: *F*_(3, 12)_ = 25.227, *P* = 0.001; anti-SCR vs. anti-miR-497: *F*_(3, 12)_ = 25.227, *P* = 0.003). Collectively, these data suggest a protective role of inhibition of miR-497 in CCl_4_-induced liver fibrosis.

## Discussion

Infection with *C. sinensis* can cause severe liver fibrosis, which may progress to liver cirrhosis and cholangiocarcinoma. Our previous studies showed that CsESPs can activate LX-2 cells, and TGF-β/Smad signaling plays a critical role in the activation of HSCs to promote the liver fibrosis caused by worm infection [7, 29]; however, the mechanism by which TGF-β/Smad is finely regulated remains ambiguous. miRNAs represent a novel regulator to prevent the production of protein at the post-transcriptional level by the degradation of target mRNA or inhibition of its translation. Several studies have shown that the expression of miR-497 is decreased in a variety of tumors [30], and it mainly inhibits the occurrence and development of tumors [31]. Studies have found that miR-497 is deficient or downregulated in a variety of malignant tumors, and downregulation of miR-497 is closely related to poor prognosis associated with tumors [32–34]. In addition, miR-497 can regulate the toll-like receptor 4/nuclear factor kappa B (NF-κB) signaling pathway, and then inhibit the expression of pro-inflammatory factor interleukin-1 beta and tumor necrosis factor alpha (TNF-α), thus playing an anti-inflammatory role [35]. Other studies have shown that miR-497, as a new regulator, is involved in TGF-β/Smad-mediated cardiac differentiation by targeting *TGFβR1* [36]. However, the expression and the roles of miR-497 in liver fibrosis are not reported. In the present study, we found that miR-497 was significantly upregulated in HSCs cells when they were activated by TGF-β_1_ or ESPs, but the expression models of miR-497 induced by TGF or CsESPs were quite different which may reflect the different capacities of CsESPs and TGF-β_1_ for the activation of HSCs. Further study showed that treatment of miR-497 inhibitor depressed the activation of HSCs and liver fibrosis both in vivo and in vitro by targeting *Smad7*, indicating that anti-miR-497 might be a promising therapeutic strategy for liver fibrosis.

Smad7 is a negative regulatory protein involved in the TGF-β/Smad signaling pathways, which act by a competitive combination with TGF-βRI to prevent phosphorylation of Smad2/3, thus inhibiting the activation of TGF-β/Smad signaling [37]. Studies have shown that several miRNAs (e.g. miR-17-5p, miR-21, miR-212) target Smad7 to moderate the activation of HSCs [38–40]. Bioinformatics analysis showed that the *Smad7* 3′ UTR contained the binding site of miR-497, and the “seed” region showed a highly conserved sequence across many different species, including humans and mice, suggesting that the mechanisms of miR-497 that moderate the expression of Smad7 might be species-independent.

To further confirm the in vitro findings, we established a liver fibrosis mouse model by the injection of CCl_4_ for 6 weeks to check whether miR-497 can regulate *Smad7* to promote liver fibrosis in vivo. CCl_4_-induced liver fibrosis is a well-accepted model not only of hepatocytes injury-induced fibrosis but also of biliary injury-induced fibrosis [41, 42]. Therefore, we used this model to investigate the mechanism underlying miR-497-regulated liver fibrosis by targeting the TGF-β/Smad signaling pathway, which may have therapeutic implications with regard to *C. sinensis* infection as well as other stimuli-induced liver fibrosis. Studies have shown that mice treated with CCl_4_ for 6 weeks (even for 4 weeks) can have severe liver fibrosis [41]. In our present study, we found that collagen deposits were significantly increased in CCl_4_-treated mice (anti-SCR group) as indicated by Masson or Sirius red staining (Fig. 5a and 5b), hydroxyproline (Fig. 5c), as well as the expression of α-SMA (Fig. 5c), suggesting that the mouse model of liver fibrosis induced by CCl_4_ was successfully established.

Recombinant adeno-associated virus serotype 8 (rAAV8) has been demonstrated as an efficient and safe therapeutic strategy for liver-related diseases, as it exhibits low immunogenicity, strong liver tropism, and long-term persistence [43, 44]. A recent study showed that *Schistosoma*-sourced sja-miR-2162 deliveried and overexpressed by rAAV8 could inhibit the expression of TGFBR3 with high efficiency, which ultimately promoted hepatic fibrosis [45]. Therefore, we adopted rAAV8 as the delivery vehicle for anti-miR-497 as well as corresponding scramble to the liver in the present study. We found that a single dose of rAAV8 could efficiently downregulate the expression of miR-497 in the liver of mice with an injection of CCl_4_, which increased the target molecule Smad7 and amelioration of liver fibrosis as indicated by the decreased expression of hydroxyproline content, α-SMA, COLI, and COLIII (Fig. 5). The present study indicated that the recombinant adeno-associated virus may represent a promising vector for gene therapy in liver fibrosis, although further studies are warranted.

## Conclusion

In conclusion, our present study provides evidence that miR-497 is involved in the pathogenesis of liver fibrosis. Our data suggest that miR-497 promotes liver fibrogenesis by targeting *Smad7* to modulate the TGF-β/Smad signaling pathway both in vivo and in vitro, which suggests that anti-miR-497 treatment represents a promising therapeutic strategy in liver fibrosis.

## Data Availability

The datasets used and/or analyzed during the current study are available from the corresponding author on reasonable request.

## Abbreviations

ANOVA: Analysis of variance
Ap: Ammonium persulphate
ALT: Alanine aminotransferase
α-SMA: Alpha smooth muscle actin
*Acta2*: Actin alpha 2, encoding α-SMA
CCL_4_: Chemokine ligand
cDNA: Complementary deoxyribonucleic acid
COLI: Type 1 collagen
*Col1a1*: Collagen type I alpha 1 chain
DMSO: Dimethyl sulfoxide
FBS: Fetal bovine serum
HSCs: Hepatic stellate cells
miR: MicroRNA
PAGE: Polyacrylamide gel electrophoresis
PBS: Phosphate-buffered saline
qRT-PCR: Quantitative real-time polymerase chain reaction
SDS: Sodium dodecyl sulfate
TGF-β_1_: Transforming growth factor beta 1
SCR: Scramble
